# Effects of supercoiling on enhancer–promoter contacts

**DOI:** 10.1093/nar/gku759

**Published:** 2014-08-14

**Authors:** Fabrizio Benedetti, Julien Dorier, Andrzej Stasiak

**Affiliations:** 1 Center for Integrative Genomics, Faculty of Biology and Medicine, University of Lausanne, 1015-Lausanne, Switzerland; 2 Vital-IT, SIB Swiss Institute of Bioinformatics, 1015-Lausanne, Switzerland

## Abstract

Using Brownian dynamics simulations, we investigate here one of possible roles of supercoiling within topological domains constituting interphase chromosomes of higher eukaryotes. We analysed how supercoiling affects the interaction between enhancers and promoters that are located in the same or in neighbouring topological domains. We show here that enhancer–promoter affinity and supercoiling act synergistically in increasing the fraction of time during which enhancer and promoter stay in contact. This stabilizing effect of supercoiling only acts on enhancers and promoters located in the same topological domain. We propose that the primary role of recently observed supercoiling of topological domains in interphase chromosomes of higher eukaryotes is to assure that enhancers contact almost exclusively their cognate promoters located in the same topological domain and avoid contacts with very similar promoters but located in neighbouring topological domains.

## INTRODUCTION

Expression of many developmentally regulated genes in higher eukaryotes requires contacts between gene promoters and distal (up to 1 Mb apart) regulatory elements known as enhancers (1,2). Enhancer–promoter interactions are mediated by transcription factors that specifically bridge them (3). Studies of interacting enhancer–promoter pairs showed that there are several ‘complementary’ classes of enhancers and their cognate promoters (4,5). The number of these classes is orders of magnitude smaller than the number of promoters and their cognate enhancer. However, in healthy cells enhancers find their cognate promoters without forming stable contacts with many other similar promoters to which they have similar affinity. How is then this fidelity of enhancer–promoter pairs assured in highly crowded nuclei where nearly all chromatin regions have the potential to interact with each other (6)? This question was partially answered with the demonstration that interphase chromosomes are divided into topological domains with the average size of ∼1 Mb (7–9). The salient feature of topological domains is that genetic loci located in the same domain interact with each other much more frequently than with loci located in different domains. In addition, it was shown that interacting enhancer–promoter pairs were almost always located in the same topological domain (10). However, the underlying mechanism responsible for enhancing contacts between loci located in the same domain is unknown. Recent studies indicated that topological domains are supercoiled due to transcription-induced supercoiling (11,12). Here we show, using Brownian dynamics simulations that when enhancer and its cognate promoter are placed in the same topological domain there is a synergism between supercoiling and enhancer–promoter affinity. That synergism ensures that enhancers spend much more time in contacts with their cognate promoters located in the same topological domain than with similar promoters but located in neighbouring topological domains. We also show regulatory advantages of large genomic separation between enhancers and their cognate promoters.

Studies of *cis-*regulatory elements of bacterial genes revealed that at least some of them can behave as enhancers capable of activating their cognate promoters over a long distance (>1000 bp) (13,14). *In vitro* experiments by Liu *et al.* showed that the action of such distally placed regulatory elements requires DNA supercoiling, whereas the same elements placed proximally act in supercoiling-independent way (13). Explaining their results, Liu *et al.* proposed that the rate of enhancer–promoter communication is the rate-limiting step during transcription activation (13). For closely spaced enhancers and promoters simple thermal fluctuations would be sufficient to bring them rapidly into a contact. Whereas, supercoiling would be needed to increase the contact rate of enhancers and promoters separated by large genomic distances (13). That proposal was based on earlier simulation studies showing that in buffers with low ionic strength, such as in buffers used for DNA storage, the elastic energy of DNA supercoiling helps to overcome electrostatic repulsion of DNA segments and thus increases the intramolecular contact rate (15–18). However, that explanation would not apply to DNA molecules under physiological conditions where electrostatic charges of DNA are neutralized. Indeed, more recent Brownian dynamics simulations showed no increase of intramolecular contact rate in supercoiled DNA with neutralized charges (18). Since the rate of contacts between enhancers and promoters is not expected to be increased by supercoiling at physiological conditions what is then the mechanism by which supercoiling stimulates enhancer–promoter interactions? We show here that supercoiling increases the fraction of time during which enhancers and promoters stay together. Importantly, this effect of supercoiling acts only when enhancers and their cognate promoters are located in the same topological domain.

## MATERIALS AND METHODS

Brownian dynamics HooMD-blue program http://codeblue.umich.edu/hoomd-blue/ (19–21) was used to simulate plasmid DNA molecules and chromatin fibres as worm-like beaded chains with bending and torsional resistance (for simulation details see (21)). Torsional resistance of our model was achieved by introducing dihedral potential that approximate axial torsional resistance of elastic continuous filaments. Our method is only precise to the first order in the bending angle (22), but it has the advantage that it does not fail for 90° bending angles (as other methods based on dihedral angle calculations frequently do). This is achieved by placing frames of references for measuring dihedral angles at middle points of the consecutive segments (21). In DNA models representing plasmids with 3000 bp the diameter of beads corresponded to 3 nm, which is the effective diameter of DNA under physiological conditions (23). The persistence length was set to 17 beads (51 nm) and torsional resistance was adjusted so that in strongly supercoiled (Δ*Lk* = −15), modelled DNA molecules ca. 80% of imposed Δ*Lk* was converted into writhe (17,24). For modelled chromatin fibres the diameter of beads was assumed to correspond to the diameter of 30 nm chromatin fibre. The persistence length of chromatin fibres was set to 60 nm, which is within the range of experimentally determined values that extend from 30 nm (25) to 150 nm (26). The partition of Δ*Lk* into ΔTw and ΔWr is not well established for chromatin. Since we investigated the effect of writhing and not of a particular Δ*Lk*, we set a high torsional stiffness giving the writhe of ∼18 for Δ*Lk* of 20. With smaller torsional stiffness we would need higher Δ*Lk* to reach the same writhe value. The standard harmonic dihedral potentials *V*(ϕ) = 0.5k (1-cos(ϕ)) with k set to 65 *k*_B_*T* or 50 *k*_B_*T* were applied for DNA and chromatin models, respectively. Taking into account the linear density of DNA in chromatin (26), each bead represents ca. 4000 bp. Modelled chromatin loops had 200 beads, which corresponds to ca. 800 000 bp and is close to the average size of topological domains (7). Beads representing enhancers and promoters were considered in a contact when their surface-to-surface distance was smaller than bead's diameter. The interaction between enhancers and promoters was modelled as truncated Lennard-Jones potential surrounding enhancer beads with the well depth ε ranging from 0 to 12 *k*_B_*T* and with r_cut set to two bead diameters (beyond the r-cut the potential is zero). The analysed configurations (6•10^5^ to 13•10^6^, depending on the equilibration time of a given system) were taken every 1000 simulation steps over simulation runs that exceeded at least 40-fold or 10-fold the equilibration time of modelled DNA molecules or chromatin loops, respectively.

## RESULTS

### Supercoiling increases the fraction of time during which enhancers and promoters stay together

**Figure 1.**
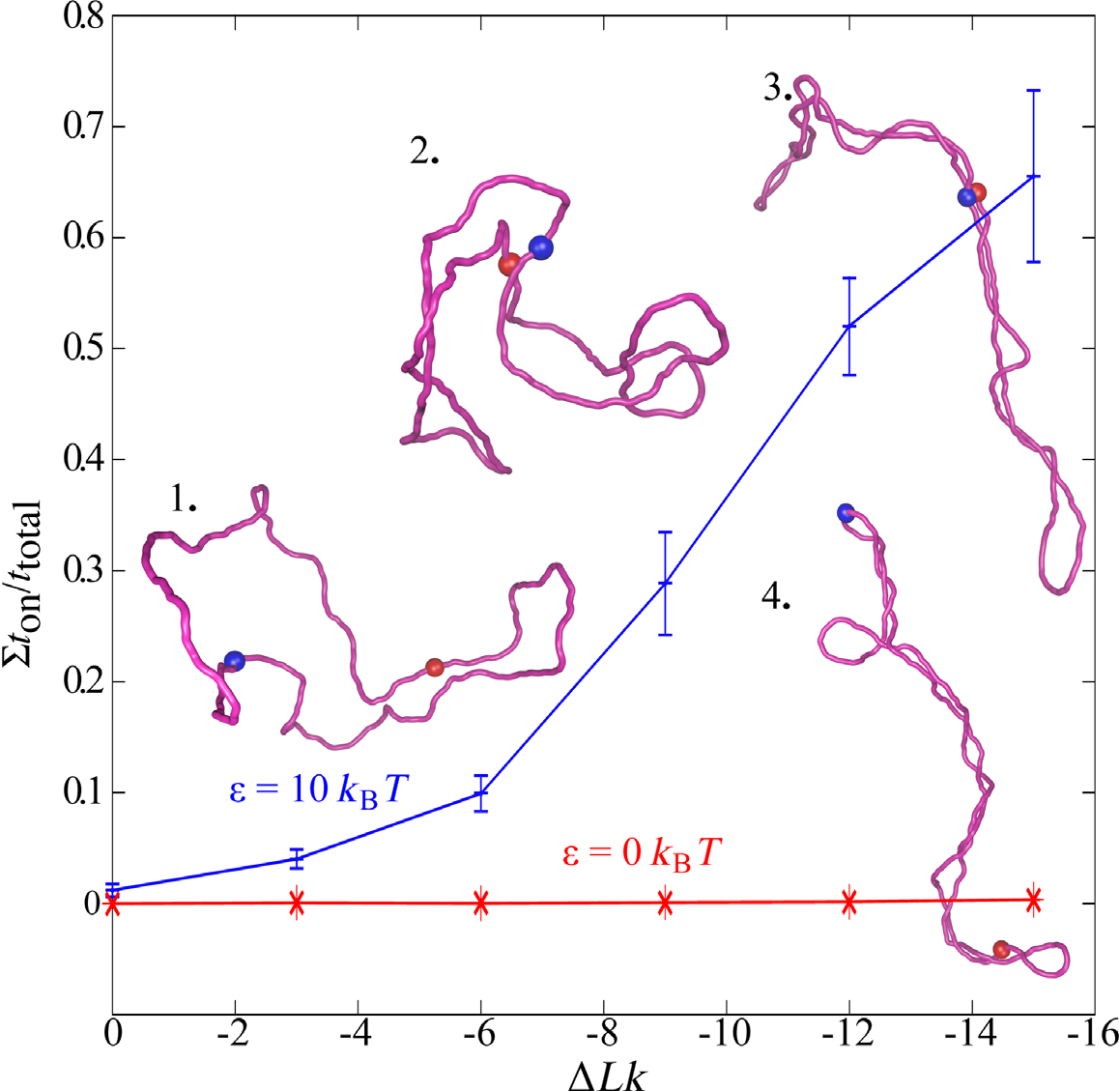
Supercoiling increases the fraction of time during which enhancer and promoter interact with each other in 3 kb long circular DNA molecules. When enhancer and promoter sites (indicated by coloured beads) show mutual affinity (here set to ε = 10 *k*_B_*T*), the fraction of time that they interact with each other (Σ*t*_on_/*t*_total_) increases with increasing magnitude of supercoiling. However, in the absence of mutual affinity between enhancer and promoter (ε = 0 *k*_B_*T*), as it would be the case of enhancers and promoters that are not supposed to bind together, supercoiling does not increase the fraction of time they interact with each other. Insets show representative simulation snapshots of corresponding DNA molecules with promoter and enhancer sites (red beads) located 180° apart on the circular map of modelled DNA molecules (snapshot 1: Δ*Lk* = 0, ε = 10 *k*_B_*T*, snapshot 2: Δ*Lk* = −9, ε = 10 *k*_B_*T*, snapshot 3: Δ*Lk* = −15, ε = 10 *k*_B_*T* and snapshot 4: Δ*Lk* = −15, ε = 0 *k*_B_*T*).

Previous simulation studies investigating the effect of supercoiling on the interactions between two sites located in the same DNA molecule treated these sites as generic polymer regions with short-range mutual repulsion (15,18). However, it is known that promoter and enhancers bind to each other due to transcription factors that specifically bridge enhancers with their cognate promoters (3). Therefore, we used Brownian dynamics simulations to investigate how the interaction between two sites located in the same DNA molecule changes with increasing DNA supercoiling and varying affinity between the two sites. In contrast to earlier studies that investigated the effect of supercoiling on the rate of juxtaposition of two sites (15,18), we measured the fraction of time during which modelled enhancers and promoters stay together. To be able to compare our simulations with these performed earlier by Huang *et al.*, we simulated first 3000 bp plasmids with various levels of supercoiling. Tested Δ*Lk* values ranged from 0 to −15, where the latter corresponds to supercoiling density σ = −0.05. Although this range did not extend to supercoiling densities observed in plasmids isolated from bacterial cells with values ranging between −0.06 and −0.075 (27), it did cover physiologically relevant range since *in vivo* supercoiling density in bacterial cells is significantly diminished by binding of histone-like proteins to DNA (28). In our simulations the effective diameter of DNA was set to 3 nm as this corresponds to the situation where the electrostatic repulsion is greatly screened such as it is the case *in vivo* (17). The lower profile in Figure 1 shows that supercoiling hardly increases the fraction of time during which two sites with no mutual affinity interact with each other. However, when two interacting sites show a mutual affinity with the strength set to 10 *k*_B_*T* (ca. 6 kcal/mol), which is an intermediate range of protein–DNA interactions that could be achieved with four to five specific hydrogen bonds (29), supercoiling greatly increases the fraction of time during which the two sites stay together (the upper profile in Figure 1). Therefore, our simulations indicate that supercoiling stabilizes interaction between cognate enhancer and promoter sites that show a mutual affinity.

### Synergism between enhancer–promoter affinity and supercoiling

**Figure 2.**
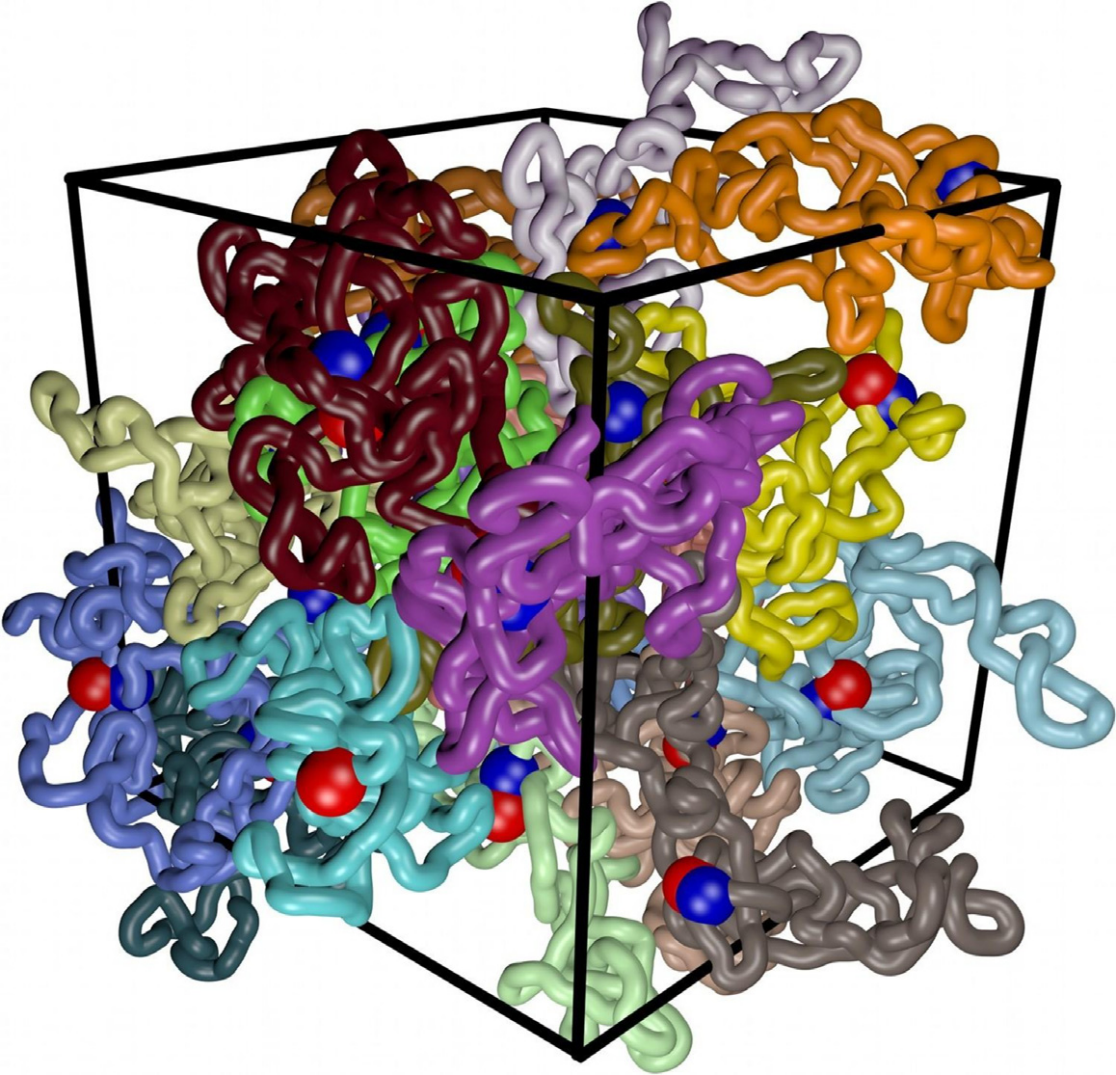
Simulation snapshot revealing highly crowded state of modelled chromatin loops. The density of modelled chromatin loops was set to 20% of volume occupation. In the shown snapshot individual supercoiled loops are each given a different colour, whereas beads corresponding to enhancers and promoters are shown as blue and red. The snapshot shows the state of the system after it has reached the equilibrium and it corresponds to the highest supercoiling (Δ*Lk* = −20) and highest enhancer–promoter affinities (ε = 12 *k*_B_*T*) investigated by us (see Figure 3). Notice that in this simulation enhancers were showing affinity only to promoters located in the same topological domain, which is different to the situation shown in Figure 4.

**Figure 3.**
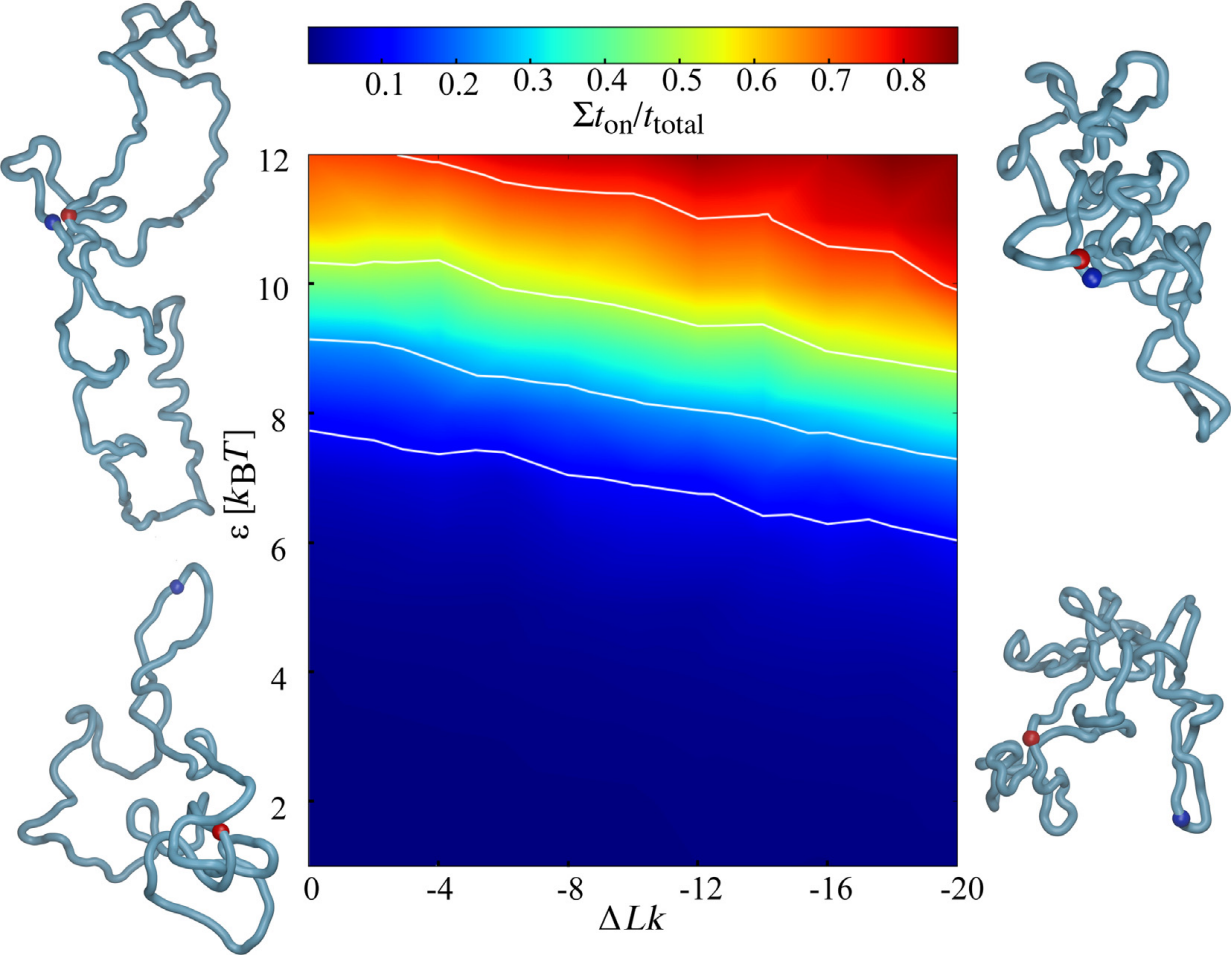
Phase diagram showing how enhancer–promoter interaction in modelled topological domains with the size of ∼800 000 bp is affected by supercoiling and enhancer–promoter affinity. Snapshots show configurations modelled topological domains take at four extreme points of the tested range of supercoiling and enhancer–promoter affinities. Enhancer and promoter regions are indicated as blue and red spheres. The actual values of data points used for cubic interpolation procedure are provided as Supplementary Data. The white isoprobability lines indicate *t*_on_/*t*_total_ values of 0.1, 0.25, 0.5 and 0.75, respectively. Non-monotonic character of the isoprobability lines results from statistical fluctuations due to the limited number of configurations that entered into the statistics.

Results presented in Figure 1 were obtained in simulations mimicking behaviour of plasmid size protein-free DNA molecules maintained in dilute solutions. The observed supercoiling-induced increase of interaction time between sites with mutual affinity can be expected to be a general phenomenon applying to any elastic filaments such as protein-free DNA or chromatin fibres. However, to perform simulations that can reflect more accurately the action of eukaryotic enhancers in further simulations, we modelled polymeric chains with characteristics of chromatin fibres that are maintained at 20% concentration, as this is believed to be the chromatin fibre concentration in interphase nuclei (30). For chromatin loops we performed simulations under periodic boundary conditions with 20 independent copies of simulated chromatin loops in the periodic simulation box. Figure 2 shows a simulation snapshot of the simulated system after it has reached the equilibrium. The shapes of individual loops are affected not only by the imposed supercoiling (Δ*Lk* = −20) but also by high crowding resulting in less elongated shapes than these observed for modelled supercoiled DNA molecules in diluted solutions (see Figure 1). We systematically investigated how changes of supercoiling and of enhancer–promoter affinity modulate enhancer–promoter interactions when they are located in the same topological domain modelled here as one closed chromatin loop (31). In our simulations we modelled enhancer and promoter sites that are spaced by ∼400 000 bp in ∼800 000 bp-long closed chromatin loop. Figure 3 shows that supercoiling and enhancer–promoter affinities act synergistically in increasing the enhancer–promoter interaction. For the tested extent of supercoiling the most interesting range of enhancer–promoter affinities spans the values from 8 to 12 *k*_B_*T*. In this range an increase of supercoiling level appreciably increases the fraction of time during which enhancer and its cognate promoter interact with each other. By modulating enhancer–promoter interaction supercoiling can have important regulatory functions in gene expression. Recent studies of interphase chromosomes in human cell lines revealed in fact that different topological domains differ in the level of supercoiling and that their supercoiling levels change dynamically (11).

### Supercoiling represses inter-domain interactions

**Figure 4.**
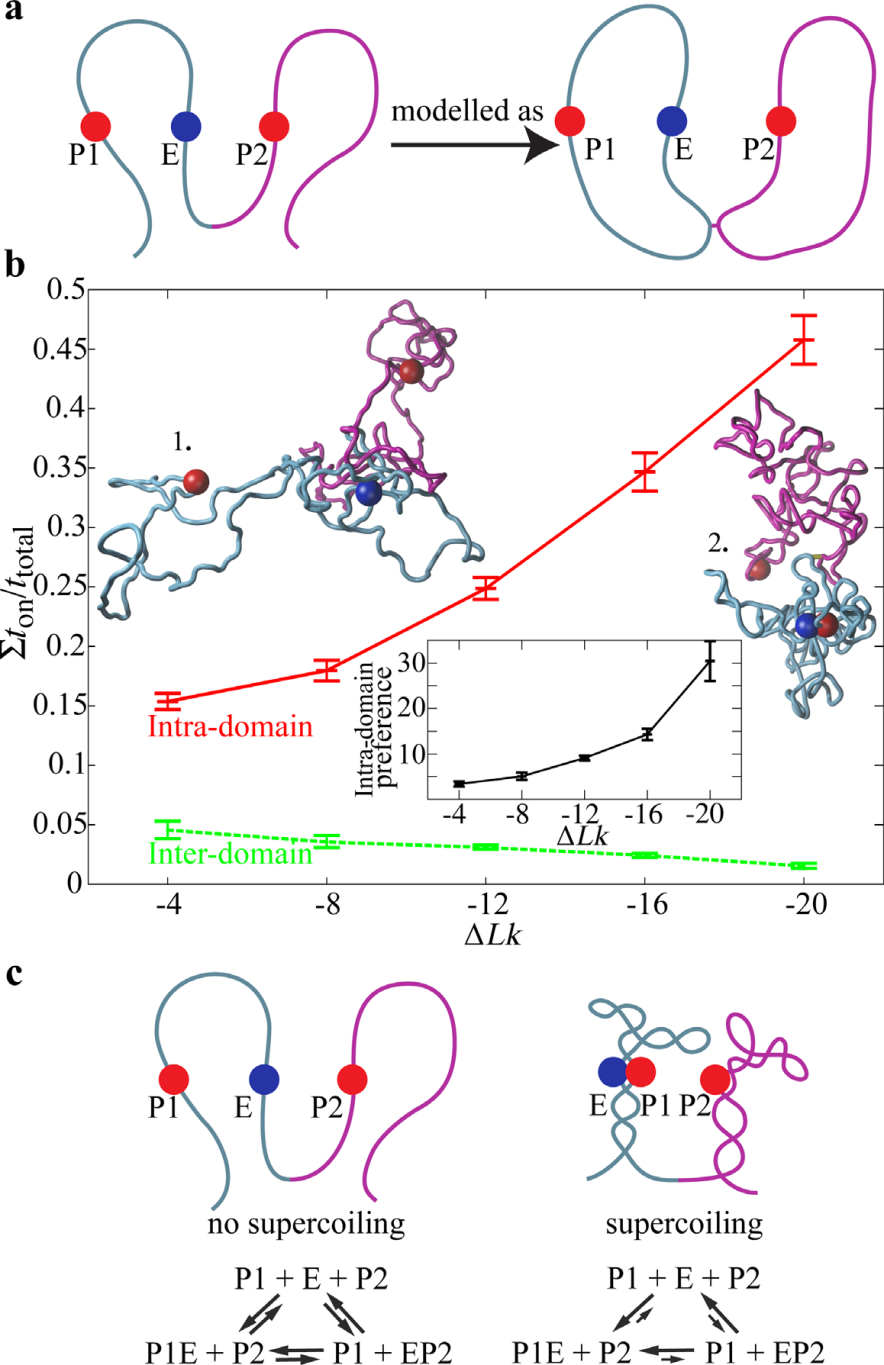
Supercoiling ensures the fidelity of enhancer–promoter interactions. (**a**) Schematic presentation of a situation where one enhancer (E) shows the same affinity to two promoters (P) that are at the same genomic distance from it but where one (P1) is located in the same and other (P2) in the neighbouring topological domain. To be able to introduce supercoiling the neighbouring chromatin loops were modelled as two closed loops tethered to each other. (**b**) Intra-domain enhancer–promoter interactions are favoured (red profile), whereas inter-domain ones are disfavoured by DNA supercoiling (green profile). The inset shows how supercoiling increases the ‘preference’ of an enhancer for intra- versus inter-domain interaction. Already without supercoiling there is a 2–3-fold preference of the enhancer to interact with the promoter located in the same domain. However, with supercoiling a 20-fold preference can easily be reached. The two simulation snapshots reflect the observation that without supercoiling (at ε = 8 *k*_B_*T*) the modelled enhancers and promoters only rarely interact with each other, whereas with increasing supercoiling one frequently observes intra-domain enhancer–promoter contacts. (**c**) ‘Ideogramic’ presentation showing the effect of supercoiling on the structure of topological domains and on the intra-domain preference of enhancer–promoter interaction.

We wanted to test whether supercoiling can have a ‘chaperone’ role and ensure that enhancers contact only (or almost only) their cognate promoters located in the same topological domains and do not get involved in contacts with promoters in other domains. To this aim we simulated a system composed of two neighbouring topological domains where an enhancer placed at the same genomic distance to two promoters has equal affinities (ε = 8 *k*_B_*T*) to them, but one promoter is in the same whereas the other in the neighbouring topological domain (see the schematic presentation in Figure 4a). As could be expected from previously presented results supercoiling increases intra-domain enhancer–promoter interactions but somewhat unexpectedly it also decreases inter-domain enhancer–promoter interactions (see Figure 4b). It is important to add here that the modelled enhancer–promoter interaction was not mutually exclusive and therefore enhancer could interact with both promoters at the same time. Inset in Figure 4 shows how the ratio between intra- and inter-domain enhancer–promoter interactions is increased by supercoiling.

Our simulations thus showed that supercoiling can stimulate or repress promoter–enhancer interactions depending on whether enhancers and promoters showing a mutual affinity are located in the same or in different topological domains (Figure 4c).

### Supercoiling is only required to stimulate distal interactions

**Figure 5.**
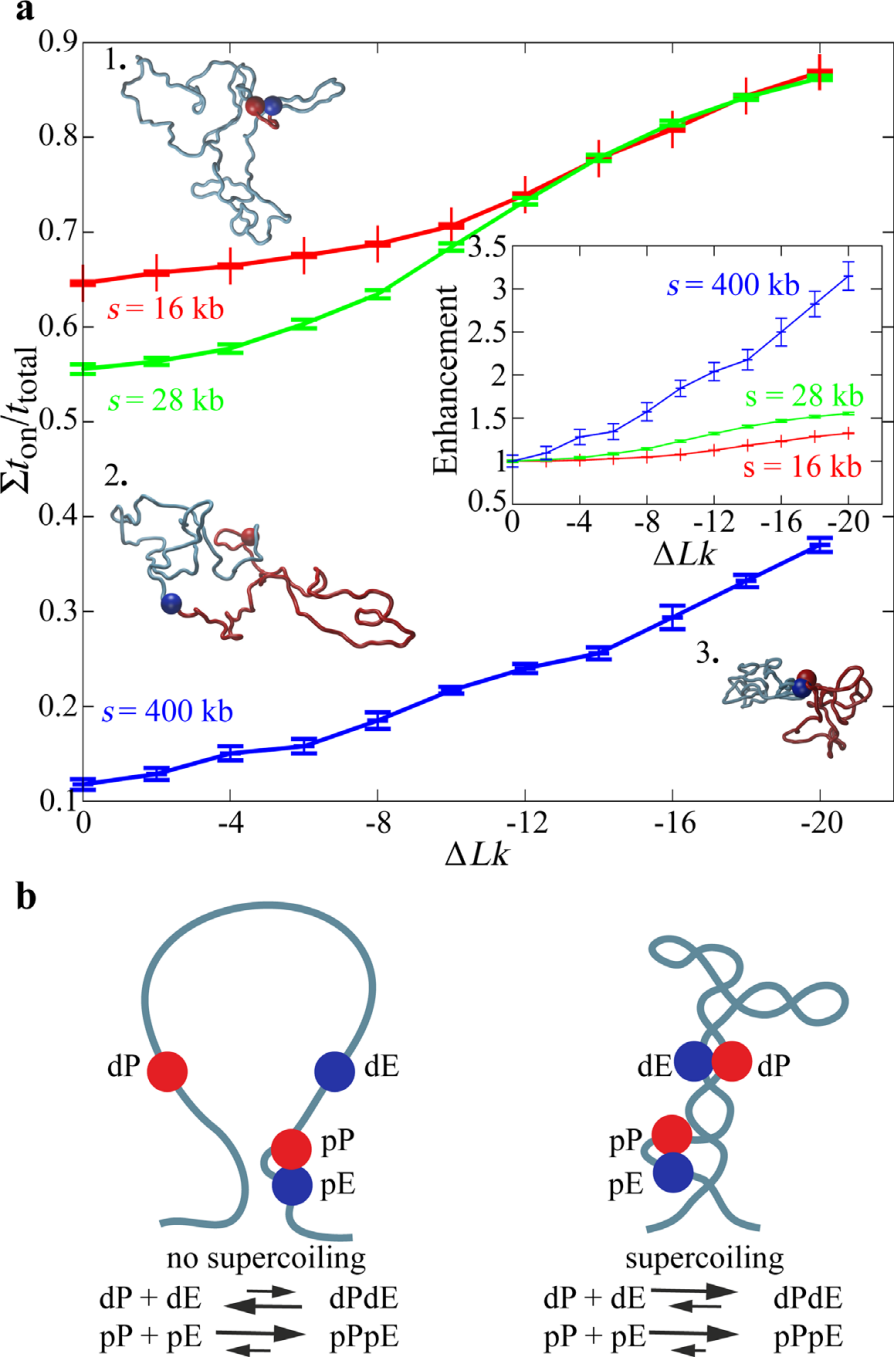
Supercoiling hardly affects intra-domain interactions between closely spaced enhancers and promoters. (**a**) Decreasing the genomic distance between enhancers and promoters located in the same topological domain increases their interaction time but diminishes the influence of supercoiling on enhancer–promoter stability (here shown for ε = 8 *k*_B_*T*). The inset shows the fold change of *t*_on_/*t*_total_ with respect to Δ*Lk* = 0 for the three tested genomic distances. For large genomic separation (*s* = 200 kb) supercoiling increases >3-fold the enhancer–promoter interaction time, whereas this enhancement index is only of ∼1.2 for small genomic separations (*s* = 16 kb). The snapshots illustrate that for small genomic separation supercoiling is not needed to stabilize enhancer–promoter interaction (1.) whereas this is not the case of large genomic separations (2., 3.). The locations of modelled enhancers and promoters are indicated with red beads. Two arcs separating modelled enhancer and promoters are indicated with different colours. (**b**) Ideogram conveying the message that the interaction between distally located enhancers and promoters (dP and dE, respectively) is strongly augmented and thus can be regulated by supercoiling. However, proximally located enhancers and promoters (pE and pP, respectively) are only marginally affected by supercoiling as they do not need supercoiling to reach relatively high *t*_on_/*t*_total_ values.

We wanted to understand why enhancers are usually distally located from their promoters. To this aim we investigated how the distance between enhancer and promoter located in the same topological domain affects the ability of supercoiling to regulate enhancer–promoter interactions. Figure 5 shows that for a moderate range of enhancer–promoter affinities (8 *k*_B_*T*) decreasing their distance from ∼ 400 kb to ∼ 16 kb made it that even without supercoiling enhancers and promoters interact for a significant fraction of time and this interaction is only slightly stimulated by supercoiling (see the inset in Figure 5a). Whereas interactions between distally located enhancers and promoters were strongly stimulated (>3-fold for the tested range of supercoiling) by increasing levels of supercoiling (Figure 5). These results suggest that large genomic distances between enhancers and promoters were selected during evolution to permit regulation of gene expression by varying level of supercoiling. However, constitutively expressed genes such as house-keeping genes should be independent of complex regulatory mechanisms involving changes of supercoiling. Indeed, house-keeping genes are not under control of enhancers but only of proximal regulatory elements (32). It is known that in bacteria changes of DNA supercoiling regulate expression of growth phase-dependent and environmentally regulated genes (33). It was proposed that supercoiling could also have regulatory function in gene expression in higher eukaryotes (34). Recent studies showed that supercoiling level is dynamically controlled in topological domains of mice chromosomes (11). We propose here that these changes in supercoiling level are important for specific regulation of contacts between cognate enhancers and promoters located in the same topological domain. Eukaryotic cells do not have DNA gyrase but transcription-induced supercoiling operates in them (12,35). Transcription of house-keeping genes that by itself is not dependent on supercoiling (Figure 5b) is sufficient to induce supercoiling in every topological domain even if the majority of the generated torsional stress is relaxed by DNA topoisomerases in a close vicinity to transcribing RNA polymerases (12). As shown in Figure 3, depending on the actual affinity between cognate enhancers and promoters different levels of supercoiling are required to stabilize their interactions and this can provide fine regulatory mechanisms switching some but not other enhancer controlled genes. Since enhancers are themselves transcribed and their expression precedes the expression of their cognate genes (36), it is possible that it is the transcription of eRNA that regulates the level of supercoiling in topological domains of interphase chromosomes.

### Supercoiling promotes rapid rebinding of dissociated enhancer–promoter complexes

**Figure 6.**
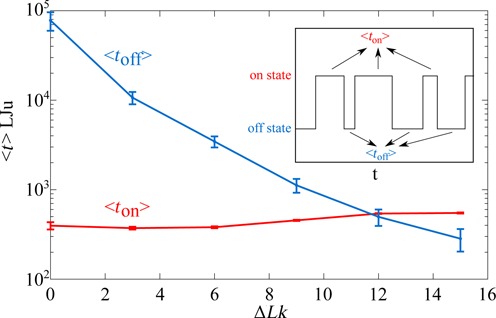
Supercoiling decreases the average duration of the off state (<*t*_off_>) without significantly changing the average duration of the on state (<*t*_on_>). Shown profiles of the evolution of <*t*_on_> and <*t*_off_> correspond to the data presented in Figure 1 where 3000 bp plasmids having two sites with the mutual affinity set to ε = 10 *k*_B_*T* were supercoiled to different extents. The inset schematically shows the telegraphic profile registering durations of individual on and off states during Brownian dynamics simulations. The on state is characterized by the distance between centres of the beads representing enhancer and promoters being smaller than 6 nm (bead diameter corresponds to 3 nm). If the distance is larger it is classified as the off state. The time is measured in reduced Lennard-Jones units used within HooMD simulation program.

It is interesting to consider the actual mechanism by which supercoiling increases the fraction of time during which enhancers and promoters stay together. To this aim we have measured the average duration time of on (<*t*_on_>) and off (<*t*_off_>) states. We observed that (<*t*_on_>) practically stays constant, whereas the <*t*_off_> decreases with increasing supercoiling (see Figure 6). These results indicate that once enhancer–promoter complex forms in supercoiled DNA its stability is hardly increased and it dissociates with k_off_ that is nearly the same as for non-supercoiled DNA. However, once such enhancer–promoter complex formed between juxtaposed, plectonemically wound segments dissociates the two partners stay for a relatively long time in close proximity since also in the dissociated state the preferred position of two opposing segments in a supercoiled molecule places them close together. The only way the previously bound segments can move further away from each other is by slithering but slithering is very slow for supercoiled molecules and its speed decreases with the density of supercoiling (37). Therefore, after the dissociation enhancers and promoters are very likely to fall back into their reciprocal attraction zones and reform the complex again. This explains then why <*t*_off_> decreases with increasing supercoiling, although from time to time enhancer and promoter sites slither away resulting in very long off states (data not shown). In non-supercoiled DNA nothing keeps dissociated promoters and enhancers in close apposition. Therefore, once they dissociate they can move away very quickly and will only very rarely fall back again into their respective zones of attractions. The above results showing a decrease of <*t*_off_> with increasing supercoiling may seem to contradict Huang *et al.* studies showing that the time to observe first contact between two sites of interest in supercoiled molecules is not affected by supercoiling (18). However, Huang *et al.* studied interactions between segments of a generic polymer where all segments only exclude each other (18). In such a situation nothing retains two sites that happened to contact each other and their <*t*_off_> just reflects unbiased dynamics of supercoiled DNA molecules or supercoiled chromatin loops. As molecules get supercoiled there are two self-compensating effects. On one hand the molecules get more compact and therefore a given segment spends a bigger fraction of time contacting other segments as compared to non-supercoiled state. On the other hand, though, the internal dynamics of the molecules decreases as slithering motion is getting slower with increasing supercoiling (37). These two self-compensating effects were invoked by Huang *et al.* to explain why the rate of inter-segmental communication does not increase with supercoiling (18). When interacting sites show significant mutual affinity the situation is changed in an interesting way. The time required for the first contact, after enhancer and promoter started to show mutual affinity through binding of relevant transcription factors, will be still long. However, once enhancer–promoter complex is formed the same slow slithering rate that was delaying first contact starts to facilitate rapid rebinding after thermally induced dissociation of enhancer–promoter complex.

## DISCUSSION

Coarse-grained polymer model helped us to reveal underlying physical principles connected to interaction between regions with mutual affinity in plectonemically supercoiled elastic polymer molecules such as DNA or chromatin fibres. Our results indicate that as long as torsional tension is able to generate loops with plectonemic structure, in which juxtaposed sites with mutual affinity bind to each other, one should expect to see very similar effects to those described here. That is, once the mutually bound sites dissociate, due to thermal agitation, supercoiling keeps them in a close proximity for a relatively long time making them much more likely to bind to each other again as compared to the situation in a non-supercoiled state. The effect we observe is most likely to be generic and thus does not depend on particular mechanical properties of DNA and chromatin. We saw the effect for two different values of persistence length, various supercoiling densities and various mutual affinities between interacting sites. The effect was similar for diluted and highly crowded conditions. Of coarse-grained polymer models are likely to miss modulating effects caused by local differences of mechanical properties of DNA or chromatin fibres. For example, reaching a juxtaposed position of a given two sites in a plectonemic superhelix may depend on the sequence effect or on different bending propensity of chromatin regions that are transcribed or not. Highly bent or highly bendable sites are likely to have the ability to organize plectonemic loops in a particular alignment frame where the apexes of supercoiled loops coincide with the position of highly bent or highly bendable sites (38). This in turn may favour or disfavour juxtapositions of certain pair of sites. In addition, the presence of several competing sites for the interaction in the same supercoiled topological domain is likely to lead to the situation where the sites with strongest mutual affinity will dictate what other sites can contact each other. Our current modelling study was not yet aimed to investigate such specific cases.

Other possible disadvantage of our coarse-grained models is their mirror symmetry making that introducing positive or negative supercoiling of the same extent produces the same results. For real DNA and chromatin the mirror symmetry is broken. However, single molecule studies of DNA have shown that at low stretching forces and supercoiling densities not exceeding these tested by us the reaction to positive and negative supercoiling is practically mirror symmetric (39). Recent *in vivo* studies of chromatin reactions to positive and negative supercoiling indicated though that positive supercoiling results in tighter superhelices of chromatin fibres than this is the case of negative supercoiling (11). Our model was not considering this difference yet. Additional possible deficiency of our modelling approach is that we have treated topological domains as closed loops whereas in reality borders of topological domains are unlikely to stick to each other (21). Therefore, real topological domains may by less free to slither than it is the case of our model and may thus be restricted in reaching certain slithering frames.

Although our work was inspired by recent studies that used modern genomic approaches to detect supercoiling in topological domains (11), one can pose the question why many earlier dedicated studies concluded that there is no unconstrained supercoiling in eukaryotic chromosomes. For example, it was shown that site-specific recombination enzymes, which action necessitates supercoiling, do not work when introduced into eukaryotic cells, whereas a mutated form of the same enzyme, which loses the dependence on supercoiling, is active in eukaryotic cells (40). Possibly the answer lies in much lower overall density of supercoiling in eukaryotic chromosomes as compared to bacterial chromosomes. The situation we have modelled here results in ca. 10 plectonemic turns for a megabase-large topological domain. Such supercoiling levels would be too low though to activate site-specific recombination systems that necessitate supercoiling to form active synaptosomes with two interwound turns completed over a distance of several hundred base pairs (40).

## CONCLUSIONS

Using Brownian dynamics simulations of coarse-grained polymer model we have shown the following:

1. Supercoiling of DNA and of chromatin fibres has the ability to increase the fraction of time during which cognate enhancers and promoters contact each other.
2. The above effect is likely to be generic since it was observed for polymer models with varying bending and torsional rigidity, varying level of supercoiling and varying mutual affinities between modelled enhancer and promoter sites. In addition, the effect was very similar for modelled polymer molecules in diluted and highly concentrated solutions.
3. The supercoiling effect operates only when the cognate enhancer and promoter sites are located in the same topological domain that is supercoiled.
4. Supercoiling-induced stimulation of enhancers-promoter interaction is most effective when the genomic distance separating enhancer and promoter is large but both are still located in the same topological domain.

## SUPPLEMENTARY DATA

Supplementary Data are available at NAR Online.

SUPPLEMENTARY DATA
