# Supplementary material for: Effects of supercoiling on enhancer–promoter contacts
Source: Nucleic Acids Res. 2014 Aug 14;42(16):10425–32. doi: 10.1093/nar/gku759 (PMC4176356; doi:10.1093/nar/gku759)
Supplement: SUPPLEMENTARY DATA [file supp_42_16_10425__index.html]

Effects of supercoiling on enhancer–promoter contacts — Effects of supercoiling on enhancer–promoter contacts — SUPPLEMENTARY DATA 

# Effects of supercoiling on enhancer–promoter contacts

## SUPPLEMENTARY DATA

**Files in this Data Supplement:**

- SUPPLEMENTARY DATA
