# Supplementary material for: Effects of supercoiling on enhancer–promoter contacts
Source: Nucleic Acids Res. 2014 Aug 14;42(16):10425–32. doi: 10.1093/nar/gku759 (PMC4176356; doi:10.1093/nar/gku759)
Supplement: SUPPLEMENTARY DATA [file supp_gku759_nar-01462-f-2014-File008.pdf]

Table S1: Datapoints for figure 2.

ΔIk	ε	<t_on/t_total>	<t_on/t_total> error
1	1	0.00268	0.00013
1	2	0.00335	0.00017
1	3	0.00383	0.00019
1	4	0.0054	0.0003
1	5	0.0135	0.0007
1	6	0.0258	0.0013
1	7	0.051	0.003
1	8	0.118	0.006
1	9	0.218	0.01
1	10	0.429	0.017
1	11	0.639	0.017
1	12	0.729	0.012
2	1	0.00245	0.00012
2	2	0.00281	0.00014
2	3	0.0045	0.0002
2	4	0.0077	0.0004
2	5	0.0149	0.0007
2	6	0.0299	0.0015
2	7	0.059	0.003
2	8	0.129	0.006
2	9	0.23	0.011
2	10	0.44	0.02
2	11	0.6104	0.0006
2	12	0.741	0.014
3	1	0.00238	0.00012
3	2	0.00379	0.00019
3	3	0.0054	0.0003
3	4	0.0086	0.0004
3	5	0.0158	0.0008
3	6	0.036	0.0018
3	7	0.07	0.003
3	8	0.15	0.007
3	9	0.275	0.013
3	10	0.43	0.015
3	11	0.618	0.015
3	12	0.767	0.01

4	1	0.00314	0.00016
4	2	0.00379	0.00019
4	3	0.006	0.0003
4	4	0.0105	0.0005
4	5	0.0147	0.0007
4	6	0.0367	0.0018
4	7	0.061	0.003
4	8	0.158	0.008
4	9	0.32	0.014
4	10	0.512	0.017
4	11	0.669	0.007
4	12	0.809	0.015
5	1	0.004	0.0002
5	2	0.0044	0.0002
5	3	0.0081	0.0004
5	4	0.0116	0.0005
5	5	0.0237	0.0012
5	6	0.05	0.002
5	7	0.096	0.005
5	8	0.185	0.009
5	9	0.335	0.013
5	10	0.54	0.03
5	11	0.691	0.007
5	12	0.822	0.011
6	1	0.0044	0.0002
6	2	0.0049	0.0002
6	3	0.0086	0.0004
6	4	0.0149	0.0007
6	5	0.0279	0.0014
6	6	0.063	0.003
6	7	0.104	0.005
6	8	0.217	0.004
6	9	0.379	0.006
6	10	0.58	0.03
6	11	0.7	0.007
6	12	0.824	0.01
7	1	0.0058	0.0003
7	2	0.0077	0.0004
7	3	0.01	0.0005
7	4	0.0167	0.0008

7	5	0.0261	0.0013
7	6	0.064	0.003
7	7	0.111	0.005
7	8	0.24	0.005
7	9	0.428	0.009
7	10	0.631	0.013
7	11	0.748	0.007
7	12	0.857	0.005
8	1	0.006	0.0003
8	2	0.0071	0.0004
8	3	0.0113	0.0006
8	4	0.0206	0.001
8	5	0.0383	0.0019
8	6	0.066	0.003
8	7	0.148	0.006
8	8	0.239	0.012
8	9	0.42	0.02
8	10	0.628	0.009
8	11	0.74	0.02
8	12	0.838	0.008
9	1	0.0056	0.0003
9	2	0.0075	0.0004
9	3	0.0133	0.0007
9	4	0.0235	0.0011
9	5	0.0431	0.0019
9	6	0.082	0.004
9	7	0.144	0.007
9	8	0.294	0.012
9	9	0.508	0.015
9	10	0.682	0.016
9	11	0.797	0.012
9	12	0.832	0.011
10	1	0.0076	0.0004
10	2	0.0087	0.0004
10	3	0.0131	0.0007
10	4	0.0226	0.0011
10	5	0.046	0.002
10	6	0.075	0.004
10	7	0.174	0.008
10	8	0.332	0.007

10	9	0.54	0.018
10	10	0.692	0.007
10	11	0.808	0.01
10	12	0.873	0.007
